# *Salmonella* in Animal Feeds: A Scoping Review

**DOI:** 10.3389/fvets.2021.727495

**Published:** 2021-11-04

**Authors:** Jan M. Sargeant, Sarah C. Totton, Mikayla Plishka, Ellen R. Vriezen

**Affiliations:** Department of Population Medicine, Ontario Veterinary College, University of Guelph, Guelph, ON, Canada

**Keywords:** *Salmonella*, feed, feed environment, livestock, poultry, evidence map, scoping review

## Abstract

The objective of this study was to describe the volume and nature of published literature on *Salmonella* in animal feeds using a formal scoping review methodology. A structured search followed by eligibility screening resulted in the identification of 547 relevant studies, encompassing studies conducted in the fields in which animal feeds are grown (15 studies), the manufacturing sector (106), during transportation (11), in the retail sector (15), and on-farm (226), with the sector not described for 204 studies. The most common study purposes were to estimate the prevalence of *Salmonella* in animal feeds (372 studies) and to identify serovars (195). The serovars that were found in animal feeds included serovars associated with human illness, with animal illness, and with serovars identified in food (livestock and poultry) intended for human consumption. There were 120 intervention studies and 83 studies conducted to evaluate potential risk factors. Within intervention and risk factor studies, there may be sufficient depth to warrant synthesis research in the areas of heat interventions, fermentation and ensiling, organic acids, season, and geographic region. Some deficiencies were identified in the completeness of reporting of key features in the relevant studies.

## Introduction

### Rationale

Non-typhoidal *Salmonella* are a leading cause of gastrointestinal disease. The global annual incidence of acute gastroenteritis in humans due to non-typhoidal *Salmonella* has been estimated at over 153 million, with an estimated 56,969 deaths (1). Approximately half (52%) of these cases are foodborne in origin (1); however, direct contact with animals is responsible for some human cases (2). In addition to the burden associated with gastrointestinal illness, data from the United States shows that the incidence of antimicrobial resistance in *Salmonella* is increasing, resulting in increased disease severity in humans and animals (3).

*Salmonella* are commonly isolated from livestock and poultry; infection in animals may be asymptomatic or may result in clinical illness of varying severity (4). Age and stress in animals are both associated with duration of fecal shedding of *Salmonella* and with severity of clinical illness. Within the farm environment, *Salmonella* can survive on surfaces as well as in water and soil (4). Thus, *Salmonella* are of concern from an animal health perspective, a public health perspective, and an environmental health perspective, thereby representing an important One Health issue.

Preventive practices are employed at the abattoir level to reduce the risk of *Salmonella* leading to foodborne illness in humans; following the introduction in 1996 of the Pathogen Reduction Hazard Analysis and Critical Controls Points (PR-HACCP) rule in processing plants in the United States, there has been an overall decline in the prevalence of *Salmonella* in meat and poultry products in that country (5). However, any progress in reducing human illness due to *Salmonella* appears to have stalled, with an overall increase in human illnesses in the United States between 2016 and 2019 (6). Additionally, control at the processing level does not address the transmission pathways of direct contact with animals or contamination of environmental sources, nor can it reduce the burden of illness in animals. Thus, there is a need to understand and control *Salmonella* along the entire farm-to-fork continuum.

Feed is a potential source of *Salmonella* exposure for livestock and poultry (7–9). In the United States, the FDA Center for Veterinary Medicine monitors trends in *Salmonella* in animal feeds and feed ingredients (10). Although the results of this surveillance suggest a decline in the prevalence of *Salmonella*-positive feed samples in the United States from 18.2% in 2002 to 8.0% in 2009 (10), *Salmonella* contamination of feed still occurs. In addition to causing illness in animals, *Salmonella* in feed can also affect human health through direct contact with contaminated feed, or from infected animals shedding *Salmonella* into human food and water sources. Understanding and controlling *Salmonella* in animal feeds involves a wide range of research approaches including diagnostic test development and validation; prevalence studies to identify which feeds are contaminated, how frequently, and when in the feed chain contamination occurs; and experimental and analytical observational designs to evaluate efficacy of interventions and to identify risk factors for contamination.

A scoping review is a type of literature review that uses transparent and replicable methods to identify all the relevant research literature on a broad topic and to categorize the extent and nature of that research (11, 12). A scoping review may act as precursor to a systematic review and/or reveal areas in which little or no primary research has been conducted (13). Although there is an extensive body of literature on *Salmonella* contamination of animal feeds, there has not been a formal scoping review conducted to categorize and describe this body of work.

### Objectives

The objective of this scoping review was to describe the primary research literature on *Salmonella* in feeds intended for livestock and poultry using formal scoping review methodology. The scoping review methods followed the framework outlined by Arksey and O'Malley (11). Reporting of this scoping review follows the Preferred Reporting Items for Systematic Reviews and Meta-Analyses Extension for Scoping Reviews (PRISMA-ScR) reporting guidelines (12), with subheadings corresponding to recommended reporting items.

## Methods

### Protocol and Registration

An *a priori* protocol was developed and published prior to the start of the review and is available at the University of Guelph Atrium: https://atrium.lib.uoguelph.ca/xmlui/bitstream/handle/10214/21331/Salmonella_in_feed_Scoping_Review_Protocol.pdf?sequence=4&isAllowed=y (accessed Aug 17, 2021). The protocol is also available at SYREAF (www.syreaf.org/protocol/). The protocol was neither formally registered nor peer reviewed.

### Eligibility Criteria

For a research study to be eligible for inclusion in this review, the following criteria needed to be met:

1) A full-text article of at least 500 words was available, written in English (although studies conducted in any country were eligible), and published during or after 1995.
2) The study involved a primary research design, including descriptive studies, experimental designs, and analytical observational designs. The number of studies conducted as *in silico* models, risk assessments, formal guideline documents, narrative reviews, systematic reviews, meta-analyses, or scoping reviews was quantified during the full-text screening stage, but these studies were not further characterized.
3) The study described an investigation of any serovar of *Salmonella* in feed intended for consumption by livestock (including fish) or poultry, or in facilities, environments, or equipment related to manufacturing, transporting, storing, or administering of feed intended for consumption by livestock or poultry.

### Information Sources

Four electronic databases were searched for relevant studies: MEDLINE® (Web of Science™), Agricola (ProQuest), CAB Direct (CABI), and Scopus. The date of the search was restricted to articles published after December 31, 1994, but no language or publication-type restrictions were applied at the search stage. A gray literature search was not conducted due to the anticipated large volume of potentially relevant research in the published literature.

### Search

The final search string as applied in MEDLINE® via PubMed is shown in Table 1; this search string also used MeSH terms and was formatted for use in the other databases. The search was conducted between October 23 and October 26, 2020. Citations identified by the database searches were uploaded into Endnote® X9 Desktop and de-duplicated using internal algorithms. The de-duplicated citations were then imported into DistillerSR® (Evidence Partners, Ottawa, ON, Canada) review management software, where additional de-duplication was conducted. Following full-text screening, a final manual de-duplication was conducted on all references that passed title/abstract screening.

**Table 1:** Search string to identify literature related to *Salmonella* in animal feeds in Medline® (via PubMed) on Oct. 23, 2020.

1	(Salmonella OR “bacterial contamination” OR “microbiological assessment” OR “microbiological quality”)
2	(“Animal feed*” OR “in-feed” OR “feeding stuffs” OR “poultry feed*” OR “Hog feed*” OR “swine feed*” OR corn OR grain* OR barley OR silage OR “crops” OR meal* OR pelleted OR pellet OR pelleting OR “dry feed*” OR “wet feed*” OR “fermented feed*” OR “feed mill*” OR feedmill* OR manufacturing OR factory OR factories OR feedstuff* OR feedingstuff* OR feed* or ration* or TMR or “total mixed ration*” or diet* or ingredient*)
3	#1 AND #2

### Selection of Sources of Evidence

Study selection was conducted using DistillerSR® and was undertaken by four individuals (the authors). Initially, eligibility was assessed using information available in the title, abstract, or meta-data available from the electronic database (language of publication). The title and abstract eligibility screening form was pre-tested by all four reviewers on 300 records. Thereafter, eligibility of each record was assessed by two reviewers working independently; agreement between the two reviewers was at the form level (include or exclude), with any disagreements resolved by consensus or in consultation with a third reviewer (JMS). The questions used for title and abstract screening were as follows:

1) Does the title or abstract describe a study investigating *Salmonella* (any serovar) in feed intended for consumption by livestock, poultry, or fish, or in facilities, environments, or equipment used for animal feeds?
2) Based on the title or abstract, is the study a primary research study (i.e., original data were collected)?
3) Is the article published in English?

Each question had response options of “yes,” “no,” or “unclear.” If the reviewers agreed that the answer to any question was “no,” the citation was not considered further. Question 2 included a response option to identify studies that used *in silico* models, or were risk assessments, guideline documents, narrative reviews, systematic reviews, meta-analyses, or scoping studies. These citations also were forwarded for full-text screening.

Due to the anticipated large number of citations identified by the search, the machine-learning ranking program available in DistillerSR® was used to assist with title and abstract eligibility screening. An initial training set of 1,000 references was used, wherein two reviewers evaluated each citation and agreed on inclusion or exclusion. Thereafter, the ranking program automatically re-ranked citations for presentation to the reviewers based on the probability that the citation would be relevant. The re-ranking occurred after every 200 records were completed with consensus by two reviewers. As an *a priori* decision, at the point where no additional eligible citations had been identified for 500 consecutively ranked references (as agreed by consensus of two reviewers), further screening was not conducted, as it was assumed that all, or almost all, eligible references would have been identified. Because more than two reviewers were evaluating references, there were 309 references with a single review conducted at the point where 500 consecutive fully assessed articles with no relevant citations was reached; eligibility screening was conducted by a second review on those 309 citations, with no additional eligible articles identified.

For citations where the response to all screening questions was “yes” or “unclear,” full-text articles were acquired through the University of Guelph's library resources. A screening form to evaluate eligibility based on information in the full text was developed in Distiller SR® and was pre-tested by all reviewers on five records. Thereafter, full-text eligibility assessment was undertaken by two reviewers independently, with any disagreements either resolved by consensus or with input from a third reviewer (JMS). The full-text eligibility screening form comprised the same three questions as the title and abstract screening form with an additional question on whether the study was available as a full text of at least 500 words. Response options were “yes” or “no.” Articles for which the response to each question was “yes” were advanced to the data charting stage. Studies that used *in silico* models, or were risk assessments, guideline documents, narrative reviews, systematic reviews, meta-analyses, or scoping studies were enumerated, but not further included in the data charting stage.

### Data Charting Process

Data charting was conducted using a form created in DistillerSR®. The form was pre-tested on five studies by all the authors. Thereafter, two reviewers working independently filled out the form for each relevant article using information provided in the title, abstract, objectives statement, methods, and results sections of the article. Conflicts were resolved by consensus or, if consensus could not be reached, with input from a third reviewer. Authors were not contacted for clarification or to obtain additional information on eligible studies.

### Data Items

The descriptive information collected from all studies included the year of publication, the country(ies) where the study was conducted, and the study design used for the component of the study related to *Salmonella* in feed (laboratory experiment, molecular study using existing isolates, clinical trial with natural disease exposure, challenge trial, single group observational study, analytical observational study, or diagnostic test evaluation). Year of publication was entered as text, and the remaining questions had fixed-choice responses.

The next question identified the sector(s) that were the source from which the animal feed-related *Salmonella* samples were collected or investigated. Reviewers selected all applicable sectors from the following options; fields used to grow animal feeds (e.g., sampling of crops in fields or grass in pastures, or of soil or irrigation water), animal feed manufacturing plant, animal feed transportation, animal feed at retail, on-farm, or not specified. The form then bifurcated, such that all additional questions were answered for each applicable sector.

The remaining questions included the species for which the feed was intended, the sources tested for *Salmonella* (including the type of feed if feed samples were the source), the *Salmonella*-related outcomes that were reported, the *Salmonella* serovars that were identified in feed from any sector, from feed, feed equipment, or the environment at the feed manufacturing stage (e.g., floor swabs, dust), or from feed or the feeding environment in the farm sector, and the purpose(s) of the *Salmonella*-in-feed component of the study. The questions had fixed-choice responses, with additional text information collected to provide further detail when the study purpose was detection or validation of diagnostic methods, risk factors for *Salmonella* prevalence or concentration, evaluation of conditions associated with survival times, or evaluation of interventions. The data extraction form is available as Supplementary Appendix 1.

After data collection was complete, a *post-hoc* descriptive categorization of the data for interventions and risk factors was undertaken to summarize the data. The data on serovars were compared to published reports on *Salmonella* serovars associated with illness in humans in the United States (US) (14) and the European Union (EU) (15), identified in food (livestock and poultry) intended for human consumption (16) and associated with illness in animals (17). These comparisons and categorizations were not described in the scoping review protocol but were added after the serovar data from the scoping review were summarized.

### Critical Appraisal of Individual Sources of Evidence

As this is a scoping review, a critical appraisal of the literature was not conducted.

### Synthesis of Results

The results of the data charting are provided descriptively, stratified by the sector(s) in which the samples were collected. Evidence gap maps provide a visual tool for illustrating the existing evidence (literature) on a topic across multiple domains (18). Evidence gap maps were created to provide additional detail on the distribution of study designs and outcomes by region and sector, by region and animal species for which the feed was intended, and by sector and animal species for which the feed was intended. In addition, evidence gap maps also were created to illustrate the study designs used by serovar and region, and serovars identified by region and species for which the feed was intended. The use of evidence gap maps was not described in the scoping review protocol but was included to better illustrate areas in which formal evidence synthesis might be warranted as well as areas in which there are research gaps.

## Results

### Selection of Sources of Evidence

**Figure 1:**
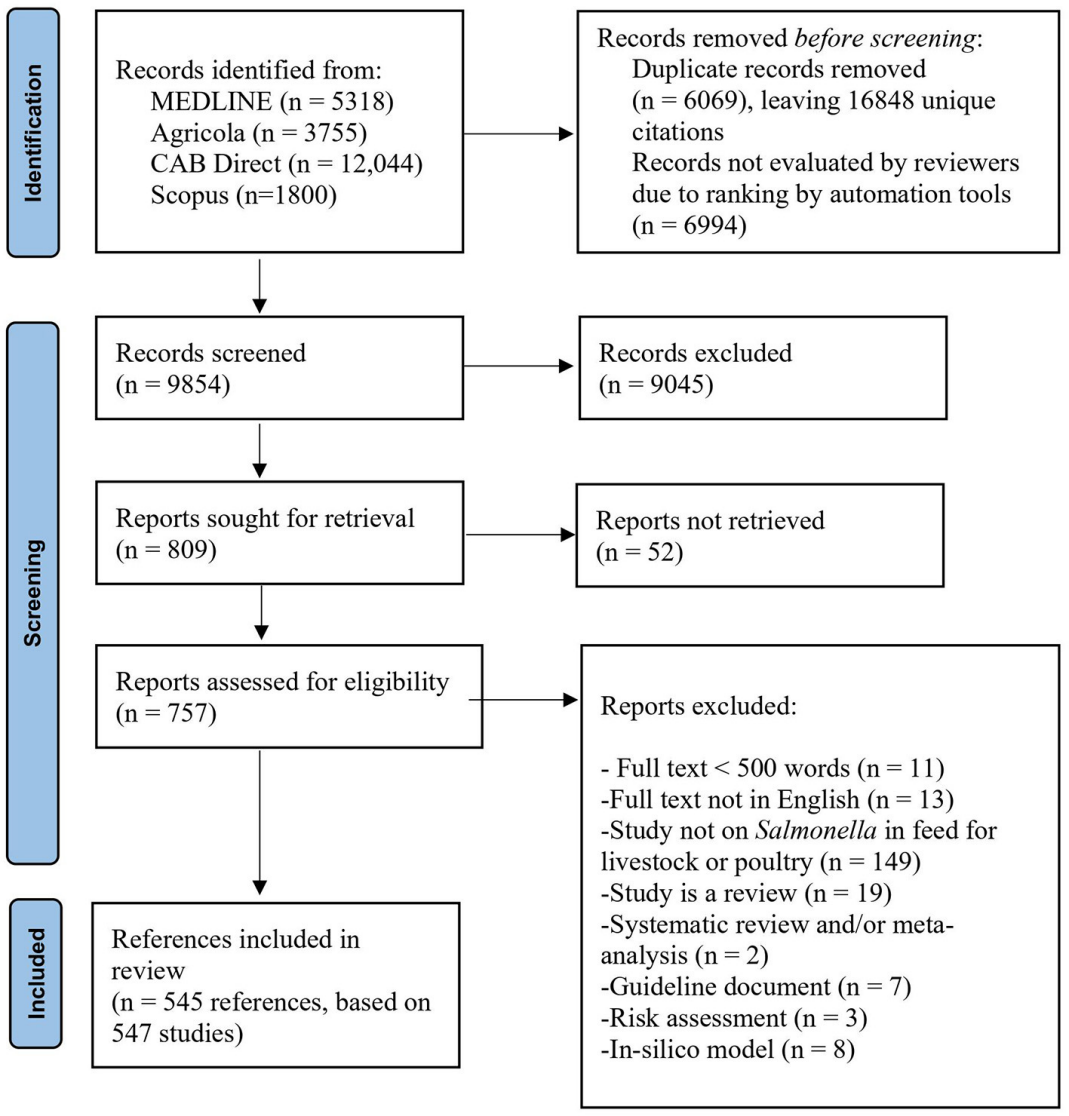
PRISMA 2020 flowchart showing the flow of citations and abstracts in a scoping review of *Salmonella* in animal feeds modified from (19).

The search identified 16,848 unique citations. After eligibility screening, there were 547 studies from 545 references included in the data charting (Figure 1). At full-text eligibility screening, 19 narrative reviews, 10 *in-silico* models, 7 guidance documents, 4 risk assessments, and 2 systematic reviews or meta-analyses were identified but not further included in the data charting (for a bibliography of these studies, see Supplementary Appendix 2).

### Characteristics of Sources of Evidence

**Figure 2:**
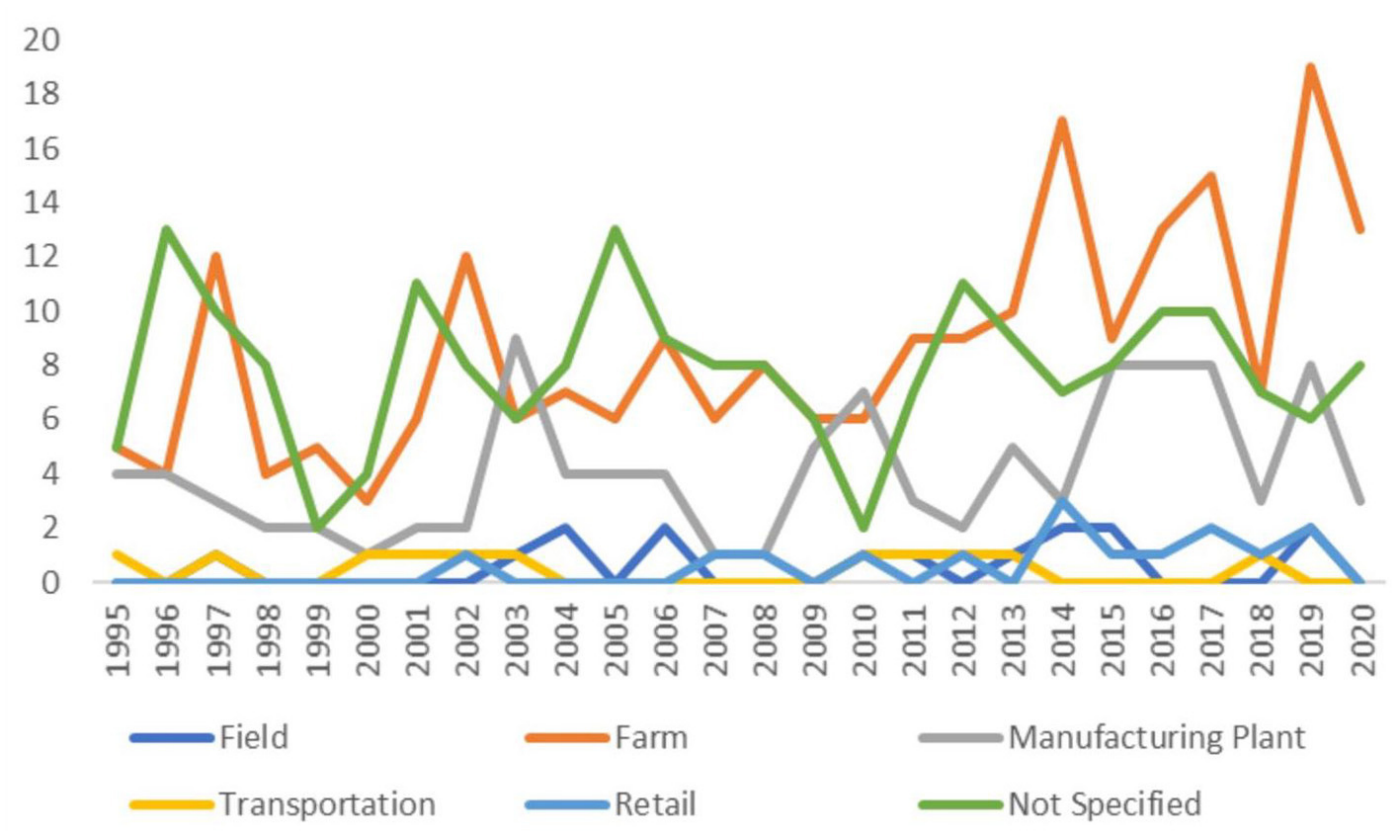
Number of studies published per year by sector of feed production as identified in a scoping review of *Salmonella* in feeds (search included publications between January 1995 and October 2020).

**Figure 3:**
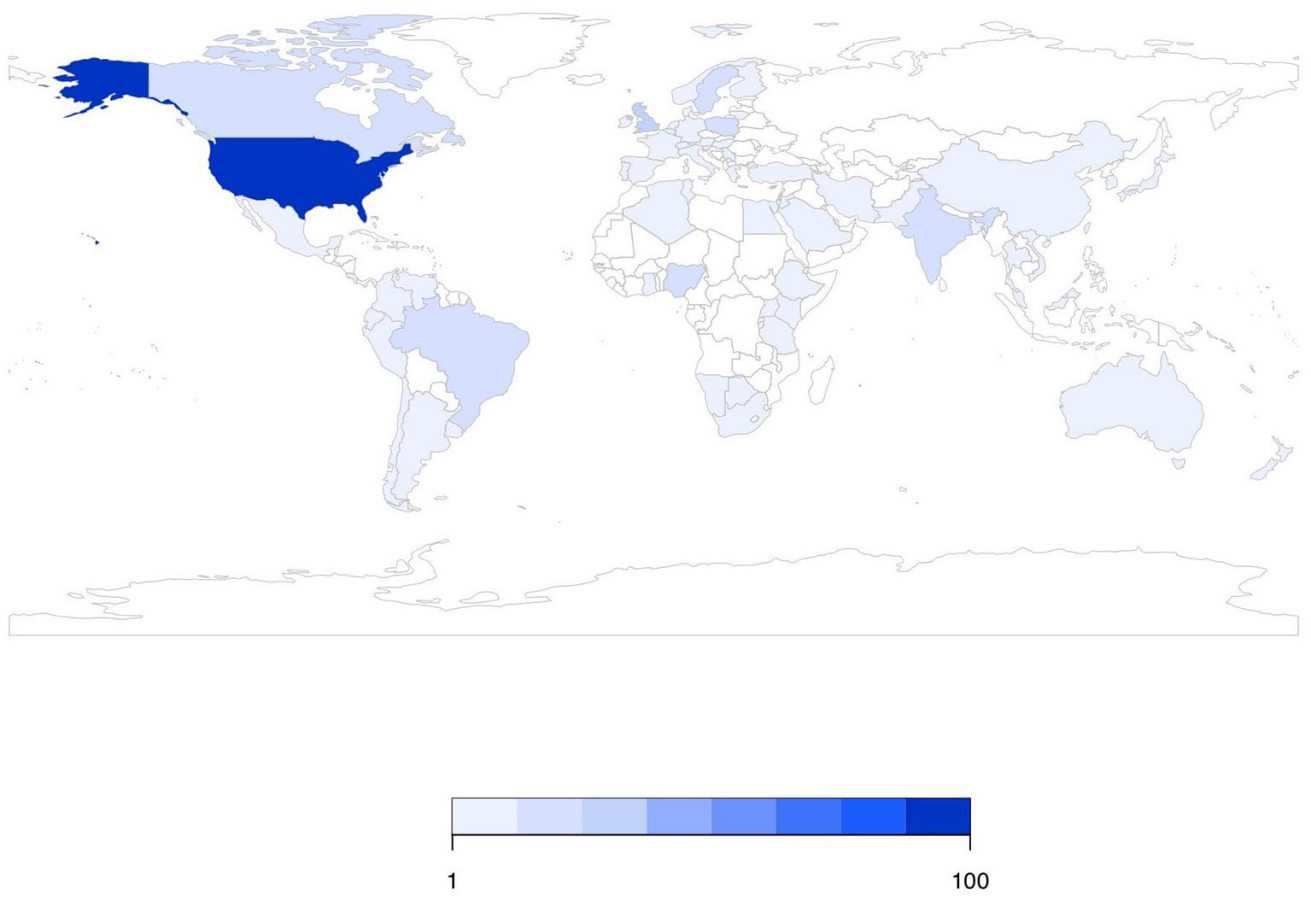
Geographic distribution of 429 studies investigating *Salmonella* in animal feeds that reported the country where the study was conducted.

There were 226 studies (41%) conducted on-farm, 106 studies (19%) conducted in plants manufacturing animal feeds, 15 studies (3%) conducted in crop fields where animal feeds were grown or pastures in which animals grazed, 15 studies (3%) conducted in the feed retail sector, and 11 studies (2%) conducted during feed transportation. The sector in which sampling was conducted was not explicitly stated in 204 studies (37%) (these percentages sum to >100% as studies could be conducted in multiple sectors). Over time, the number of publications did not appear to change dramatically (Figure 2). Figure 3 shows the geographic distribution of 429 studies; the most common country in which studies were conducted was the United States, followed by Great Britain, Brazil, Nigeria, and Poland. The country where the study was conducted was not reported in 118 studies (26%).

### Critical Appraisal Within Sources of Evidence

Not applicable, as this is a scoping review.

### Results of Individual Sources of Evidence and Synthesis of Results

The included studies encompassed feed intended for a wide range of species, although the species for which the feed was intended was not reported in over one-third of the studies (189/547; 35%) (Table 2). For many studies in which species was not reported, the sector in which the sampling was conducted also was not reported (113/189; 60%). Numerically, the feed sampled from the manufacturing plant, transportation, retail, and farm sectors was most commonly feed intended for swine and poultry.

**Table 2:** Summary of results for 547 studies investigating *Salmonella* in animal feeds by sector of animal feed production.

	**Number of studies^a^**					
	**Field/Pasture** **(*N* = 15)**	**Plant** **(*N* = 106)**	**Transport** **(*N* = 11)**	**Retail** **(*N* = 15)**	**Farm** **(*N* = 226)**	**Not specified** **(*N* = 204)**
**Species for which the feed was intended^a^**						
Not reported (*N* = 189)	6	53	2	5	10	113
Poultry or chickens, not further specified (*N* = 105)	–	23	1	8	24	49
Swine (*N* = 103)	–	29	2	2	44	26
Broiler chickens (*N* = 84)	–	17	2	2	43	20
Laying hens (*N* = 76)	–	13	3	3	42	15
Cattle, not further specified (*N* = 42)	4	8	–	3	14	13
Dairy cattle (*N* = 37)	2	3	–	–	28	4
Turkeys (*N* = 28)	–	3	1	1	20	3
Beef cattle (*N* = 19)	2	2	–	–	12	3
Fish/Shellfish (*N* = 19)	–	4	1	2	7	5
Sheep (*N* = 15)	2	2	–	–	3	8
Ruminant, not further specified (*N* = 11)	1	3	1	–	1	5
Goats (*N* = 8)	1	–	–	1	2	4
Other farmed poultry (*N* = 5)	–	–	–	1	2	2
Domestic ducks (*N* = 3)	–	–	–	–	2	1
Buffalo/Bison (*N* = 1)	–	–	–	–	1	–
Meat rabbits (*N* = 1)	–	–	–	–	1	–
**Source(s) tested for** ***Salmonella*** ^**a**^						
Feed (*N* = 537)	11	101	10	15	199	201
Feed environment (*N* = 81)	8	30	1	–	40	2
Feed equipment (*N* = 41)	–	20	2	–	16	3
Not specified (*N* = 6)	1	–	–	–	–	4
Other (*N* = 5)	1	4	–	–	1	–
**Study design**						
Single group observational (descriptive only) (*N* = 169)	5	36	6	6	98	18
Analytical observational (*N* = 139)	5	35	4	3	74	18
Laboratory study with experimental manipulation (*N* = 101)	1	8	–	2	10	80
Diagnostic test assessment (*N* = 74)	–	10	–	4	14	46
Molecular characterization, previously obtained isolates (*N* = 66)	–	15	1	–	12	38
Trial with natural exposure to *Salmonella* (*N* = 23)	1	2	–	–	17	3
Challenge trial in natural setting (*N* = 5)	3	–	–	–	1	1
**Purpose of the** ***Salmonella*** **in feed component of the study^a^**						
Estimating prevalence of *Salmonella* (*N* = 372)	9	68	10	9	187	89
Determining serovars of *Salmonella* (descriptive) (*N* = 195)	–	45	8	1	100	41
Evaluation of interventions to reduce *Salmonella* (*N* = 121)	3	19	–	–	36	63
Molecular characterization (descriptive) (*N* = 115)	1	28	2	2	49	33
Determining antimicrobial resistance (descriptive) (*N* = 111)	–	16	4	2	55	34
Estimating concentration of *Salmonella* (*N* = 98)	6	15	1	2	22	52
Identifying risk factors for prevalence or concentration (*N* = 85)	5	19	4	1	36	20
Development/Validation of detection methods (*N* = 82)	–	9	–	5	17	51
Estimating survival time for *Salmonella* (*N* = 54)	–	4	–	1	8	41
Outbreak investigation with animal feed component (*N* = 20)	–	5	–	–	12	3
Comparing serovars between sectors (*N* = 16)	–	11	1	–	4	–
Comparing prevalence of *Salmonella* between sectors (*N* = 15)	–	11	–	–	3	1
Comparing molecular characteristics between sectors (*N* = 10)	–	4	–	1	2	3
Comparing antimicrobial resistance between sectors (*N* = 7)	–	4	–	–	1	2
Evaluation of conditions associated with survival times (*N* = 4)	–	1	–	–	1	2
Development or validation of surveillance methods (*N* = 4)	–	1	–	1	2	–
Evaluation of linkages between *Salmonella* in feed and human illness (*N* = 3)	–	1	–	–	1	1
***Salmonella*** **outcomes reported^a^**						
Serovar(s) in food/Food environment/Food equipment (*N* = 292)	5	64	7	6	97	113
Prevalence/Proportion positive (*N* = 283)	5	62	7	9	135	65
Molecular characteristics (*N* = 93)	–	24	2	3	34	30
Concentration (*N* = 92)	6	12	1	3	16	54
Reported *Salmonella* absent, no denominator (*N* = 81)	–	8	1	2	33	37
Antimicrobial resistance (*N* = 68)	–	15	2	1	28	22
Survival time (*N* = 59)	3	3	–	1	9	43
Reported *Salmonella* present, no denominator (*N* = 55)	–	8	–	2	25	20
Reported number of positive samples, no denominator (*N* = 18)	–	4	–	1	6	7
Odds ratios/Risk ratios (*N* = 5)	–	3	–	–	2	–
Incidence (*N* = 2)	–	–	–	–	2	–
Results were combined among sample types (*N* = 102)	2	11	3	1	63	22
None of the above (*N* = 18)	–	3	–	–	3	12
No results presented (*N* = 3)	–	1	–	–	2	–

a *More than one response could be selected for a study, so the total may exceed the total number of studies characterized*.

The source(s) tested for *Salmonella* was identified in all except 6 studies (1%); although feed was the most commonly sampled source (537/547; 98%), the environment was sampled in 8/15 studies (53%) in the field sector, equipment or the environment were sampled in 50/106 studies (47%) in manufacturing plants and in 3/11 studies (27%) in the transportation sector, and, in the farm sector, the feed environment or equipment used for handling feed was sampled in 56/226 studies (25%) (Table 2). The high proportion of studies in which feed was sampled reflects the scoping review objective and the literature search conducted.

There were 251 reports of feeds sampled in which no description of the feed type was provided (Table 3). Across sectors, feed types sampled to test for *Salmonella* included poultry litter intended as animal feed (21 studies), complete feeds or commercial feeds (64 studies), roughage or silage (35 studies), supplements including premix or mineral mix (25 studies), pelleted feeds (49 studies), and single-ingredient feeds (262 studies) (Table 3). Of the feeds that were reported, single-ingredient feeds were the most commonly sampled feed type in all sectors. Roughages and poultry litter intended as animal feeds were only sampled on-farm and in studies where the sector was not specified. Within single-ingredient feeds, the feedstuffs were further categorized *post-hoc* based on the Canadian Food Inspection Agency's National Feed Inspection Program categories (https://inspection.canada.ca/animal-health/livestock-feeds/inspection-program/salmonella-monitoring-program-for-livestock-feeds/eng/1514931465271/1514931465993) (accessed Aug 17, 2021). These categories comprised animal by-products (141 studies), grains and oilseeds (138 studies), plant protein products (79 studies), recycled food products (19 studies), grain by-products (10 studies), and other (45 studies). Grains and oilseeds, and animal by-products were the most commonly sampled categories within the single-ingredient category within each sector. The specific feedstuffs (as described by the authors) that were assigned to each of these subcategories, by sector, are available in Supplementary Appendix 3. As this is a scoping review, study results were not extracted, so the prevalence of *Salmonella* in these feed types is not reported here, and it is unknown which of these sample types were found to contain *Salmonella*.

**Table 3:** Types of animal feeds sampled to investigate *Salmonella* across sectors of animal feed production from 537 studies.

**Category of animal feed^a^**	**Plant** **(*N* = 11 studies)**	**Transport** **(*N* = 10)**	**Retail** **(N = 15)**	**On-Farm** **(*N* = 199)**	**Not specified** **(*N* = 201)**
No description of feed type sampled (*N* = 251)	36	4	2	124	85
Single ingredient^b^ (*N* = 262)	79	5	9	51	118
Animal by-products (*N* = 141)	39	5	4	19	74
Grains and oilseeds (*N* = 138)	50	1	5	28	54
Plant protein products (*N* = 79)	26	1	3	8	41
Other (*N* = 45)	8	1	2	13	21
Recycled food products (*N* = 19)	6	0	0	5	8
Grain by-products (*N* = 10)	2	0	0	3	5
Pelleted feeds (*N* = 69)	29	0	1	20	19
Complete feed, commercial feed, mixed feed (*N* = 64)	13	1	9	14	27
Roughage, silage (*N* = 35)	0	0	0	20	15
Supplements, premix, mineral mix (*N* = 25)	5	1	1	7	11
Poultry litter intended as animal feed (*N* = 21)	0	0	0	14	7

a *Totals sum to more than the number of studies, as sampling of multiple categories of animal feed could be described within a single study*.

b *Subcategories of single ingredients used classification scheme based on Canadian Food Inspection Agency's National Feed Inspection Program (https://inspection.canada.ca/animal-health/livestock-feeds/inspection-program/salmonella-monitoring-program-for-livestock-feeds/eng/1514931465271/1514931465993) (accessed Aug. 21, 2021)*.

For studies in which the sector of feed production was reported, the most common study design was a single group observational study [6/11 (55%) for transport, 98/226 (43%) for on-farm, 5/16 (40%) for field, 6/15 (40%) for retail, and 36/106 (34%) for manufacturing plant], although analytical observational designs also were common in the manufacturing plant sector (35/106; 33%) and on-farm (74/226; 33%) (Table 2). Of the studies in which the sector of feed production was not reported, most were laboratory studies (80/204; 39%), followed by diagnostic test assessment (e.g., determination of analytical or epidemiological sensitivity and/or specificity of a diagnostic test) (46/204; 23%) and molecular characterization studies (38/204; 19%). To further explore the types of study designs used, evidence gap maps illustrating study design by region and sector, by region and species for which the feed was intended, and by sector and species for which the feed was intended can be accessed at: https://salmonella-in-animal-feeds.github.io/instructions.html (study design tab) (accessed Aug 17, 2021). In these on-line evidence gap maps, specific information on the distribution of study designs for combinations of region, sector, and species for which the feed was intended can be accessed by hovering over the bubbles on the diagrams. Observations from these gap maps include a lack of experimental studies from some regions (e.g., Oceania), most of the field trials were conducted in the on-farm sector across most regions, and single group observational studies were distributed across most regions and species for which the feed was intended, and across most sectors.

The most common study purpose across all sectors was estimating the prevalence of *Salmonella* in feed, feed equipment, or the feed environment; estimating prevalence was at least one of the study purposes for 10/11 studies (transportation; 91%), 187/226 (farm; 83%), 68/106 (manufacturing plant; 64%), 9/15 (field; 60%), 9/15 (retail; 60%), and 89/204 (not specified; 44%) studies (Table 2).

Determining serovars of *Salmonella* also was a common study purpose, particularly for studies conducted in the manufacturing plant and on-farm sectors [45 (23%) and 100 (51%)], respectively, of the 195 studies evaluating serovars (Table 2). In some instances, serovars were reported as an outcome for a study but not as a study purpose; examples include outbreak investigations of a specific serovar and diagnostic test assessment studies in which a specific serovar was used. In studies that investigated *Salmonella* serovars, a wide range of serovars were reported across sectors: field-level (12 serovars identified), manufacturing plant (281 serovars), transportation (85 serovars), retail (13 serovars), farm (98 serovars), unspecified (134 serovars). A complete list of the *Salmonella* serovars investigated in each sector is available in Supplementary Appendix 4 and the 10 serovars most frequently investigated within each sector are listed in Table 4. Summed across sectors, the 10 most frequently investigated serovars were Typhimurium (reported in 148 studies), Enteritidis (99 studies), Senftenberg (86 studies), Mbandaka (68 studies), Infantis (65 studies), Montevideo (54 studies), Agona (53 studies), Anatum (43 studies), Livingstone (34 studies), and Tennessee (34 studies). Table 4 also identifies whether each serovar identified in feed among the 20 most common serovars associated with human illness in the United States (US) (14) or in the European Union (EU) (15), among the five most common serovars identified in four foods (livestock or poultry) intended for human consumption (16), or among the five serovars most commonly associated with illness in four food animal species (17). A complete list of the 20 most common serovars associated with human illness in the US and EU, the five serovars most found in each species at processing, and the five serovars most commonly associated with illness in each animal species is provided in Supplementary Appendix 5. Thirteen of the 33 serovars (39%) commonly associated with human illness in the US or EU were amongst those most frequently investigated in feed. Nine of the 13 serovars (56%) associated with illness in animals were amongst the most frequently investigated serovars in animal feed. Finally, 11 of the 17 serovars (65%) commonly identified in food intended for human consumption were amongst the most frequently identified serovars in animal feed.

**Table 4:** Ten *Salmonella* serovars most frequently investigated in animal feeds, feed equipment, or feed environment by sector of animal feed production (number of studies in which serovar was reported, alphabetic listing for tied results).

**Sector of animal feed production**					
**Field**	**Plant**	**Transport**	**Retail**	**On-farm**	**Unspecified**
Typhimurium (2) ^H−EU,H−US,An,Food^	Senftenberg (37) ^An^	Infantis (4) ^H−EU,H−US,An,Food^	Enteritidis (3) ^H−EU,H−US,An,Food^	Typhimurium (32) ^H−EU,H−US,An,Food^	Typhimurium (75) ^H−EU,H−US,An,Food^
Typhimurium DT140 (1)	Typhimurium (37) ^H−EU,H−US,An,Food^	Livingstone (4)	Newport (3)^H−EU,H−US,An,Food^	Enteritidis (26) ^H−EU,H−US,An,Food^	Enteritidis (47) ^H−EU,H−US,An,Food^
Anatum (1) ^H−US,Food^	Agona (30)^H−EU,H−US,An,Food^	Mbandaka (4)	Cubana (2)	Senftenberg (18) ^An^	Senftenberg (25) ^An^
Derby (1) ^H−EU,An,Food^	Mbandaka (30)	Oranienburg (4) ^H−US^	Typhimurium (2) ^H−EU,H−US,An,Food^	Mbandaka (17)	Agona (23)^H−EU,H−US,An,Food^
Durham (1)	Montevideo (26)^H−US,An,Food^	Senftenberg (4) ^An^	Bareilly (1) ^H−EU^	Infantis (14) ^H−EU,H−US,An,Food^	Infantis (21) ^H−EU,H−US,An,Food^
Kedougou (1)	Infantis (25) ^H−EU,H−US,An,Food^	Montevideo (3) ^H−US,An,Food^	Choleraesuis (1)	Derby (11) ^H−EU,An,Food^	Mbandaka (15)
Mbandaka (1)	Enteritidis (23) ^H−EU,H−US,An,Food^	Ohio (3)	Heidelberg (1) ^An^	Worthington (11)	Montevideo (15) ^H−US,An,Food^
Montevideo (1) ^H−US,An,Food^	Anatum (20) ^H−US,Food^	Orion (3)	Infantis (1) ^H−EU,H−US,An,Food^	Anatum (10) ^H−US,Food^	Tennessee (15)
Newport (1) ^H−EU,H−US,Food^	Schwarzengrund (18) ^Food^	Rissen (3)	Javiana (1)^H−US^	Kentucky (10) ^H−EU,An,Food^	Livingstone (14)
Rissen (1) Senftenberg (1) ^An^ Stanley (1)^H−EU^	Livingstone (16) Tennessee (16)	Schwarzengrund (3) ^Food^ Tennessee (3)	Kentucky (1)^H−EU,An,Food^ Mbandaka (1) Montevideo (1) ^H−US,An^ Senftenberg (1)^An^	Montevideo (8) ^H−US,An,Food^	Anatum (12) ^H−US,Food^

H−EU *Among the 20 most common serovars associated with human illness in the European Union (15)*.

H−US *Among the 20 most common serovars associated with human illness in the United States (14)*.

An *Among the five most common serovars per species associated with illness in animals in the United States (17)*.

Food *Among the five most common serovars reported in food intended for human consumption in the United States (16)*.

Additional information on the distribution of study designs by serovars and region, and for common serovars identified by region, species for which the feed was intended, and sector can be found in evidence gaps maps available at: https://salmonella-in-animal-feeds.github.io/instructions.html (accessed Aug. 17, 2021).

There were comparatively fewer studies in which the study purpose was to test hypotheses such as comparing prevalence, antimicrobial resistance, serovars, or molecular characteristics between sectors, identifying risk factors for prevalence or concentration of *Salmonella*, or evaluating interventions to reduce *Salmonella* (Table 2). There was a total of 120 intervention studies: 63 studies (53%) were conducted in unspecified sectors, 36 (30%) were conducted on-farm, 20 (17%) were conducted in feed manufacturing plants, 3 were conducted in the field sector (3%), and no studies were conducted in the transportation or retail sectors (1 study evaluated interventions in 2 sectors). The study designs employed in the intervention studies were observational studies (27/120; 23%), experimental studies in a laboratory setting (73/120; 61%), deliberate *Salmonella* challenge studies in a natural setting (i.e., not a laboratory) (2/120; 2%), and field trials with natural exposure to *Salmonella* (18/120; 15%).

The interventions were categorized *post-hoc* into management strategies (7 studies), cleaning and disinfection (18 studies), crop strategies (2 studies), physical feed treatments (46 studies), chemical processes (24 studies), and feed additives (34 studies) (Table 5). Within these categories, there was a range of specific interventions, the majority of which were evaluated in a single study. The five most common interventions evaluated were heat or steam (21 studies), organic acids (15 studies), ensiling (13 studies), fermentation (10 studies), and stack depth for poultry litter (9 studies).

**Table 5:** Summary of 120 intervention studies of *Salmonella* in animal feeds, feed equipment and feed environment.

**Intervention categories**	**Number of studies by sector**	**Examples of specific interventions evaluated**
Physical feed treatments (*N* = 46)	Not specified: 19 Farm: 15 Plant: 12	Heat or steam (21), stack depth (poultry litter) (9), radiation (4), hydrothermal (2), pelleting/extrusion (2), drying (2), high-pressure processing (2), pasteurization (1), flocculation (1), drying (1), rendering (1), storage time (1), wrapping layers (hay) (1), radiofrequency heating (1), microwaving (1), radioactive uranium (1)
Feed additive (*N* = 34)	Not specified: 28 Farm: 4 Plant: 2	Organic acids (15), formaldehyde (4), essential oils (4), medium-chain fatty acids (2), antimicrobials (2), sodium butyrate (1), nitro-treatment (1), probiotics (7), bacteriophage (1), novacid (non-antibiotic) (1), bacitracin methylene disalicylate (1), pre-biotics (1), food waste (1), LAPg media (1), ammonia (1), copper sulfate (1), ethyl alcohol (1)
Chemical processes (*N* = 23)	Not specified: 17 Farm: 4 Plant: 2 Field: 1	Ensiling (13), fermentation (10), aerobic activation (1), black soldier fly composting (1)
Cleaning and disinfection (*N* = 18)	Farm: 11 Plant: 4 Not specified: 3	Acidic electrolyzed water (2), removal of deposits and adhered materials (1), steam (1), formaldehyde (1)
Management strategies (*N* = 7)	Farm: 6 Plant: 1	HACCP program (1), good farming practices (1), starling control (1), separation of hospital and maternity pens (1), depopulation (1), depletion (1), eradication procedure (1)
Crop strategies (*N* = 2)	Field: 2	Fertilizer type (dairy manure vs. chemical fertilizer) (1), irrigation type (borehole water vs. treated sewage effluent) (1), harvest technique (first cutting vs. second cutting) (1)

There were 83 studies evaluating risk factors for *Salmonella* in animal feeds (Table 6), with one study evaluating risk factors in two sectors. Observational study designs were employed for the majority of risk factor studies (67/83; 90%), with laboratory experiments (4/83; 5%), deliberate *Salmonella* challenge studies in a natural setting (1/83; 1%), and field trials with natural exposure to *Salmonella* (3/83; 4%) also used. The most common category of risk factor evaluated in the manufacturing plant, transportation, farm, and unspecified sectors was characteristics of the feed. Environmental risk factors such as temperature and climate were evaluated in 16 studies across sectors.

**Table 6:** Summary of 83 risk-factor studies (number of studies) evaluating *Salmonella* in animal feeds, feed equipment and feed environment across sector of animal feed production.

**Sector of animal feed production**					
**Field (*****N*** **= 5)**	**Plant (*****N*** **= 19)**	**Transport (*****N*** **= 4)**	**Retail (*****N*** **= 1)**	**Farm (*****N*** **= 36)^a^**	**Unspecified (*****N*** **= 20)**
Field characteristics (2)	Plant characteristics (9)	Transportation characteristics (1)	Retail site characteristics (0)	Farm characteristics (21)	
Season, climate or weather (3)	Season or climate (4)	Season or climate (0)	Season or climate (1)	Season or climate (8)	Season or climate (0)
Geographic region (2)	Geographic region (4)	Geographic region (2)	Geographic region (0)	Geographic region (7)	Geographic region (1)
Crop characteristics (0)	Feed characteristics (19)	Feed characteristics (3)	Feed characteristics (0)	Feed characteristics (14)	Feed characteristics (15)
	Equipment characteristics (0)	Equipment characteristics (0)	Equipment characteristics (0)	Equipment characteristics (0)	Equipment characteristics (0)
Other (0)	Other^d^ (3)	Other (0)	Other (0)	Other^b^ (10)	Other^c^ (7)

a *Column totals may exceed the total number of studies for a sector because studies could evaluate more than one risk factor*.

b *Age (2) and type of chickens (2), location from which feed was sampled (1), manure treatment on soil and forage (1), week of production (1), antimicrobial use (1), source (1), poultry production and biosecurity (i.e., number of years working on the farm, staff working on different farms, use of rodent traps, visitor registration, water supply system, shoe and vehicle disinfection barriers) (1), Salmonella surveillance (1), flock cycle (1), presence of starlings (1), year of study (1), restriction of visitor entry into chicken houses (1), downtime (1)*.

c *Year (5), airflow in housing and location of infected bird (1), Alphitobias beetles (1)*.

d *Day of the week (1), pelleting temperature (1), year (1)*.

Diagnostic test accuracy was assessed in 81 studies (Table 7). In most of these studies, the sector in which the samples were collected was not specified (58/81; 72%) although diagnostic test accuracy studies also were conducted in the farm sector (19/81; 23%), manufacturing sector (11/81; 14%), and retail sector (6/81; 7%). Studies involved either naturally contaminated or deliberately contaminated samples, and the test types included culture-based tests, DNA-based tests, enrichment methods, and other approaches.

**Table 7:** Summary of 81 detection-method studies of *Salmonella* in animal feeds, feed environment and feed equipment across sector of animal feed production.

	**Number of studies by sector of feed production^a^**			
	**Unspecified** **(*****N*** **= 51)**	**Farm** **(*****N*** **= 17)**	**Manufacturing** **(*****N*** **= 9)**	**Retail** **(*****N*** **= 5)**
Samples naturally contaminated (*N* = 49)	24	15	8	3
Samples deliberately contaminated (*N* = 44)	34	4	3	3
Culture-based test(s) (*N* = 57)	36	10	8	3
DNA-based test(s) (*N* = 49)	29	13	3	5
Enrichment method(s) (*N* = 29)	19	4	5	1
Other (*N* = 24)	18^e^	1^d^	4^b^	1^c^

a *Studies may have utilized both naturally contaminated and deliberately contaminated samples, evaluate multiple diagnostic test types, or include more than one sector. Therefore, the total numbers may exceed the number of studies*.

b *ELISA (2), Technique to enhance recovery of Salmonella (1), immunochromatographic test (1)*.

c *Pre-PCR processing strategies (1)*.

d *Modified 1–2 Test™ System (1)*.

e *ELISA (5), immunomagnetic separation technique for enhanced recovery of Salmonella (2), Reveal® 2.0 Group D1 Salmonella lateral flow immunoassay (1), Dynabeads Anti-Salmonella System (1), Polymacron Enzyme Immunoassay System (1), polymyxin-cloth enzyme immunoassay (1), biochemical methods (1), indirect impediometry (1), “one-step Salmonella isolation test” (1), modified immunodiffusion (1), Loop-Mediated Amplification/ISO 6579-Based Method (1), Neogen Reveal® Test (1), hybridization sensor (1), slide agglutination (1)*.

Twenty *Salmonella* outbreak studies included an animal feed component and three studies (one each in the manufacturing plant, on-farm, and not specified sectors) evaluated linkages between *Salmonella* in animal feed and human illness (Table 2).

The pattern for study design and study purpose(s) were consistent with the pattern for the *Salmonella*-related outcomes reported, with the majority of the outcomes being descriptive (including prevalence, concentration, molecular characteristics, antimicrobial resistance patterns, and serovar identification) (Table 2). Across all sectors, there were 250 studies in which the results were combined among sample types (e.g., serovar results were presented, but the results combined feed with other sources), or the number of positive samples or the presence or absence of *Salmonella* was reported without a denominator.

Evidence gap maps illustrating outcome categories by region and sector, by region and species for which the feed was intended, and by sector and species are available at: https://salmonella-in-animal-feeds.github.io/instructions.html (accessed Aug. 17, 2021). With the exception of the field sector, studies in which serovars, molecular characteristics, and antimicrobial resistance were included as outcomes were distributed across regions and across species for which the feed was intended.

## Discussion

### Summary of Evidence

The results of this scoping review reveal a breadth of literature related to *Salmonella* in animal feeds. The relevant studies identified in this review represented feed intended for a wide variety of food animal species, although feed intended for swine and poultry was most frequently studied. Most studies sampled animal feed, although equipment and the feed environment also were investigated. The *Salmonella* serovars that were identified in animal feeds included serovars commonly associated with human and animal illness and also with food (livestock and poultry) intended for human consumption, highlighting the complexity and the One Health nature of *Salmonella* in animal feeds. A range of study designs and study purposes were identified in the included studies, encompassing descriptive studies, intervention and risk factor studies, and diagnostic test development and evaluation. However, there were few studies evaluating the role of animal feed in animal health (20 outbreak studies) or in human health (3 studies).

A depth in the research literature in some areas provides insight into topics that may be amenable to formal systematic review. For instance, the large number of studies that estimated prevalence in feed types, particularly grains and oilseeds, and in animal by-products, may be sufficient to allow a systematic review to estimate a summary prevalence in these feeds and potentially to explore reasons for heterogeneity (differences among studies). The evidence gap maps provide additional detail on studies designs and outcomes available in the literature by region, species for which the feed was intended, and by sector. This information may be helpful for researchers considering conducting a systematic review in a specific sector, geographic region, or species for which feed is intended, or for understanding the distribution of these factors as possible sources of heterogeneity in a more broadly structured systematic review.

Similarly, systematic reviews of intervention efficacy may be possible for heat interventions, fermentation and ensiling, and organic acids. It is noteworthy, however, that the study designs employed for the intervention studies were variable and included observational designs and experimental designs in the laboratory, using deliberate *Salmonella* challenge models, and field trials. This range of experimental designs presents challenges for synthesis research on interventions and has implications for conducting meta-analyses (the quantitative component of a systematic review whereby results of multiple studies are combined to estimate a summary intervention effect size) (20). Within the experimental designs, laboratory experiments and deliberate disease challenge models provide proof-of-concept for the efficacy of an intervention whereas field trials provide a higher level of evidence for the efficacy of an intervention under real-world conditions (21). There is empirical evidence that deliberate disease induction trials may reach different conclusions, even if conducted in the same species (22). Thus, it may not be appropriate to combine the results of these different experimental approaches in a meta-analysis. Observational studies of intervention efficacy can be included in a meta-analysis, but the potential of confounding bias for the specific topic area should be considered when determining whether this is appropriate (23).

The finding that observational study designs were common for risk factor studies is not surprising, given that not all risk factors are amenable to investigator allocation. Although there was less replication of risk factor evaluations, there may be potential to conduct systematic reviews on risk factors related to season or climate, or among geographic regions.

The results of this scoping review also provide an indication of areas in which there may be gaps in knowledge. Notably, most of the interventions that were described were evaluated in only one study. Trials on the same research question often give different results, because of nuanced differences in the populations, interventions, comparison groups, and outcome metrics and measurements, as well as because of statistical and biological variability (24–26). Therefore, decisions on whether to implement an intervention should be based on a synthesis of the results of multiple trials. Thus, there is a need to replicate intervention assessments to provide a robust understanding of the true efficacy of an intervention or importance of a risk factor.

### Limitations

#### Limitations of the Data

The studies identified in this scoping review investigated *Salmonella* in animal feed primarily in feed manufacturing plants or at the farm-level. The research literature also was dominated by studies conducted in the United States, which may not be representative of the depth and breadth of literature from other regions.

All but two of the studies measured prevalence or concentration at one point in time as the outcome measure for *Salmonella*, rather than sampling over time to determine incidence outcomes. This information is useful for knowing where *Salmonella* exists but does not provide information on when contamination occurred. It also means that evaluations of intervention efficacy will target control of *Salmonella*, rather than prevention of contamination. While this is useful information, studies evaluating where contamination occurs on the feed production continuum would be useful to identify where preventive strategies may best be applied.

Reporting of key issues was deficient in many studies; the sector in which the feed samples were collected or sourced was not reported for over the third of the studies (204/547), the species for which the feed was intended also was not reported in over a third of the studies (188/547), and the country or region was not reported for 118 studies. In addition, there were studies in which the results were combined across sample types, or where presence or absence of information for *Salmonella* was reported but without providing a denominator to allow the calculation of prevalence. Missing or incomplete information limits the ability of the reader to interpret the results as well as the usefulness of the information for secondary data purposes, such as systematic reviews to synthesize data on prevalence, risk factors, or intervention efficacy across multiple studies or as inputs to risk assessments. Reporting guidelines are available to provide guidance for authors for writing articles on the results from trials [the REFLECT statement; (27, 28) and from observational studies (the STROBE-Vet statement; (29, 30)]. Authors, journal editors, and peer-reviewers may find these guidelines a valuable resource for improving the quality and comprehensiveness of reporting the results of research studies.

#### Limitations of the Review

This scoping review focused on *Salmonella* in animal feeds; it is noteworthy that this is only one possible source of *Salmonella* to livestock and poultry, and livestock and poultry are not the only sources of *Salmonella* in humans. A scoping review approach documents the extent and nature of the research literature on *Salmonella* in animal feeds and is not intended to provide information on the relative importance of animal feeds to the large issue of animal and human illness due to *Salmonella*. Thus, the scoping review results should be interpreted in this context.

This scoping review was restricted to publications in the English language, which may mean that the results do not reflect the body of literature on *Salmonella* in feed that is available in other languages. The number of records excluded at full-text screening because they weren't in English was small (13/757); however, the search terms were English, thus the actual extent to which language bias may exist could not be evaluated. In addition, the selection of studies was limited to the past 25 years and thus may not be representative of the literature on this topic that was conducted prior to this time.

The scoping review encompassed a broad topic area, and therefore the search terms also were broad, resulting in a large volume of articles to screen for eligibility. For this reason, machine learning was used in this study to facilitate the identification of eligible studies at the title and abstract screening level. This was conducted using an automated machine learning tool within the DistillerSR software that re-ranks citations for presentation to the reviewers after every 200 citations with inclusion decisions. A heuristic stopping approach was used, whereby reviewers stopped screening after there were an *a priori* defined number of citations screened without identifying an eligible article (31). It is possible that some relevant articles remained in the dataset of citations which were not screened by human reviewers. Not achieving 100% recall (sensitivity) in the screening process arguably is less essential in a scoping review where the purpose is descriptive, compared to a systematic review, where the purpose is aggregation of results from multiple studies (32). Finally, a gray literature search was not conducted, and it is therefore possible that there exist relevant reports published outside of the journals indexed in the electronic databases search.

## Conclusions

Using scoping review methods, a breadth and depth of literature on *Salmonella* in animal feeds was identified, with studies conducted in fields where animal feeds are grown as well as in the feed manufacturing, transportation, retail, and on-farm sectors. The studies included descriptive studies, primarily estimating prevalence or identifying *Salmonella* serovars, intervention and risk factor evaluations, and diagnostic test evaluations. The results of this scoping review provide insight into areas in which there may be a sufficient body of literature to warrant formal systematic reviews (e.g., heat interventions, fermentation and ensiling, and organic acids), as well as identifying gaps in our knowledge (e.g., low number of studies conducted outside of the United States and lack of replication of studies on specific interventions and risk factors). The review also identified deficiencies in reporting of critical information in studies, which limits their interpretability as well as the ability to include the studies in evidence syntheses such as systematic reviews and meta-analyses.

## Data Availability Statement

The original contributions presented in the study are included in the article/Supplementary Material, further inquiries can be directed to the corresponding author.

## Author Contributions

JS and ST developed the review protocol. JS conducted the literature search. MP created the evidence maps. JS wrote the first draft of the manuscript. All authors were involved in eligibility screening, data extraction, analysis, interpretation of the results, reviewed, and approved the final manuscript.

## Funding

Funding for this study was provided by the Pew Charitable Trusts (GR-000009656). Representatives from the Pew Charitable Trusts had the opportunity to review the study protocol to provide input on the breadth of the data being collected. They also reviewed a copy of the manuscript prior to submission for publication.

## Author Disclaimer

The views expressed herein are those of the author(s) and do not necessarily reflect the views of The Pew Charitable Trusts.

## Conflict of Interest

The authors declare that the research was conducted in the absence of any commercial or financial relationships that could be construed as a potential conflict of interest.

## Publisher's Note

All claims expressed in this article are solely those of the authors and do not necessarily represent those of their affiliated organizations, or those of the publisher, the editors and the reviewers. Any product that may be evaluated in this article, or claim that may be made by its manufacturer, is not guaranteed or endorsed by the publisher.
