# Supplementary material for: Salmonella in Animal Feeds: A Scoping Review
Source: Front Vet Sci. 2021 Nov 4;8:727495. doi: 10.3389/fvets.2021.727495 (PMC8600132; doi:10.3389/fvets.2021.727495)
Supplement: Supplementary file 1 [file Data_Sheet_1.PDF]

Supplementary materials:

Appendix 1: **Data extraction form (with some data extraction options truncated) for the scoping review of *Salmonella* in animal feeds.**

1. What is the year of publication?
2. In which country was the study conducted? (select all that apply)

    Not reported

    USA

    Canada

    China

    Viet Nam

    (additional countries listed with option for reviewer to add new country if necessary)

3. What study design was used for the *Salmonella* and feed component (based on actual, if different from authors' label)?

    Laboratory study (experimental)

    Challenge trial in non-laboratory setting

    Clinical / field trial with natural disease exposure

    Single group observational

    Analytical observational

    Molecular studies (e.g. categorization from isolate banks, antimicrobial resistance)

    Diagnostic test evaluation

4. In which setting(s) was the source of the feed-related *Salmonella* that were investigated? (Select all that apply)

    Field used to grow animal feeds

    Feed manufacturing plant

    Feed transportation (e.g., feed trucks)

    Animal feed at retail

    On-farm (including farmed fish sites)

    Not specified

Answer the following questions specifically for the setting of fields used to grow animal feeds.

5. For which species was the feed intended? (Select all that apply)

Dairy cattle Beef cattle Buffalo / bison Cattle, not further specified Sheep Goats Small ruminant, not further specified Ruminants, not further specified Swine Farmed cervids Livestock, not further specified Broiler chickens Laying hens Poultry, not further specified Chickens, not further specified Domestic ducks Turkeys Rabbits raised for meat Other farmed poultry Fish / shellfish Not reported

6. Which source(s) were tested for *Salmonella*? (Check all that apply)

    Not specified

    Crop in field

    Pasture or grass

    Soil

    Irrigation water

    Animal feces present in the field

    Broiler litter intended for spreading on field

7. *Salmonella* outcomes reported (Check all that apply)

    Prevalence / proportion positive

    Incidence

    Concentration

    Survival time

    Molecular characteristics

    Serovar(s) present in food / food environment / food equipment

    None of the above

    Odds ratios / risk ratios

    Hazard ratios

    No results presented

    Results were combined among sample types

    Reported number of positive samples without a denominator

    Reported "*Salmonella* present" (or +) without a denominator

    Reported "*Salmonella* absent" (or -) without a denominator

8. Which *Salmonella* serovars were investigated or reported? (Select all that apply)

    Not specified / combined across sample types / *Salmonella* spp.

Senftenberg

Mbandaka

Tennessee

Montevideo

(Additional serovars listed, with option for reviewers to add if not in list)

9. What was the purpose of the *Salmonella* in feed component of the study? (Select all that apply)

Development of validation of methods for detection of *Salmonella*
If yes, were samples contaminated or deliberately contaminated / inoculated?

Naturally infected

Deliberately inoculated

Both natural and inoculated

If yes, what type of method was used?

Culture-based

DNA-based

Enrichment methods

Other

Development or validation of surveillance methods

Estimating prevalence of *Salmonella*

Estimating concentration of *Salmonella*

Estimating survival time of *Salmonella*

Determining antimicrobial resistance

Determining serovars present

Molecular characterization (descriptive)

Comparison of antimicrobial resistance patterns between sources

Comparison of serovars between sources

Comparison of molecular characterization between sources

Risk factors for prevalence or concentration of *Salmonella*
If yes, risk factor(s) evaluated? (Select all that apply)

Geographic region

Season, climate or weather

Field characteristics

Crop characteristics

Other

Evaluation of conditions associated with survival times
If yes, condition(s) evaluated? (Select all that apply)

Temperature

Rainfall

Humidity

Soil type

Crop type

Evaluation of interventions to reduce *Salmonella*
If yes, describe the intervention [text box]

Outbreak investigation

Evaluation of linkage to illness in humans

Comparison of prevalence between sources

Estimating incidence of *Salmonella*

Answer the following questions specifically for the setting of feed manufacturing plant / feed mill.

10. Same as Q5

11. Which source(s) were tested for *Salmonella*? (Check all that apply)

not specified

feed

equipment

environment

other

12. Same as Q7

13. Same as Q8

14. Same as Q9, with response option for risk factors and associations with survival times as below

Which risk factor(s) were evaluated? (Select all that apply)

Geographic region
Season, climate
Plant characteristics
Feed characteristics
Equipment characteristics
Other

Which condition(s) associated with survival times were evaluated (Select all that apply)

Temperature
Feed type
Hazard Analysis Critical Control Points (HACCP)
Soil type
Crop type
Surface type

Answer the following questions specifically for the setting of feed transportation.

15. Same as Q5
16. Same as Q6
17. Same as Q7
18. Same as Q8

19. Same as Q9, with response option for risk factors as below

Which risk factor(s) were evaluated? (Select all that apply)

Geographic region

Season, climate

Transportation characteristics

Feed characteristics

Equipment characteristics

Other

Answer the following questions specifically for the setting of animal feed at retail.

20. Same as Q5
21. Same as Q6
22. Same as Q7
23. Same as Q8

24. Same as Q9, with response option for risk factors as below

Which risk factor(s) were evaluated? (Select all that apply)

Geographic region

Season, climate

Retail site characteristics

Feed characteristics

Equipment characteristics

Other

Answer the following questions specifically for the setting of on-farm (including aquatic).

25. Same as Q5
26. Same as Q6
27. Same as Q7
28. Same as Q8

29. Same as Q9, with response option for risk factors as below

Which risk factor(s) were evaluated? (Select all that apply)

Geographic region

Season, climate

Farm characteristics

Feed charactxeristics

Equipment characteristics

Other

Answer the following questions specifically for setting not specified.

30. Same as Q5
31. Same as Q6
32. Same as Q7
33. Same as Q8

34. Same as Q9, with response option for risk factors and associations with survival times as below

Which risk factor(s) were evaluated? (Select all that apply)

- Geographic region
- Season, climate
- Feed characteristics
- Equipment characteristics
- Other

Which condition(s) associated with survival time were evaluated? (Select all that apply)

- Temperature
- Humidity
- Feed type
- Surface type

Appendix 2: **Bibliography of narrative reviews, systematic reviews, guidance documents, risk assessments and *in-silico* models related to *Salmonella* in animal feeds.**

Narrative Reviews:

Alexander TW, Stanford K, McAllister TA. On-farm mitigation of enteric pathogens to prevent human disease. In: Krause D, Hendrick S, editors. *Zoonotic Pathogens in the Food Chain.* CABI Publishing (2010). p.140-66.

Binter C, Straver JM, Häggblom P, Bruggeman G, Lindqvist PA, Zentek J, et al. Transmission and control of *Salmonella* in the pig feed chain: a conceptual model. *Int J Food Microbiol.* (2011) 145:S7-S17. doi: 10.1016/j.ijfoodmicro.2010.09.001.

Conchello L. *Salmonella* control throughout the "Poultry Feed Chain". *World Poultry* (2011) 27:14-5.

Davies RH, Hinton MH. *Salmonella* in animal feed. In: Wray C, Wray A, editors. *Salmonella in Domestic Animals.* CABI Publishing (2000). p.285-300. doi: 10.1079/9780851992617.0285.

European Food Safety Authority (EFSA). Report of the Task Force on Zoonoses Data Collection on the availability of molecular typing methods for *Salmonella, Campylobacter*, verotoxigenic *Escherichia coli, Listeria monocytogenes* and *Staphylococcus aureus* isolates from food, animals and feedingstuffs in European Union Member States (and in some other reporting countries). *EFSA Journal* (2009) *7*:272r. doi: 10.2903/j.efsa.2009.272r.

Gopo JM, Baros E, Kent AV. Developing and marketing of a *Salmonella*-specific DNA diagnostic kit in Southern Africa. In: Serageldin I, Persley G, editors. *Biotechnology and Sustainable Development: Voices of the South and North.* CABI Publishing (2003). p.187-90.

Grace D, Lindahl JF, Kang'ethe EK, Harvey JJ. Detecting and preventing contamination of dairy cattle feed. In: vanBelzen N, editor. *Achieving Sustainable Production of Milk. Volume 2: Safety, Quality and Sustainability.* Cambridge, UK: Burleigh Dodds Science Publishing Limited (2017). p.95-116. doi: 10.19103/AS.2016.0006.16.

Häggblom P. 2012. How does Sweden control *Salmonella* before it enters the food chain?. In: Hoorfar J, editor. *Case Studies in Food Safety and Authenticity.* Cambridge, UK: Woodhead Publishing (2012) p.198-205.

Hanson DL, Ison,JJ, Malin KS, Webb, HE. *Salmonella White Paper*. Beef Industry Food Safety Council (BIFSCo) (2016). 52p.

Liebana E, Hugas M. Assessment of the microbiological risks in feedingstuffs for food-producing animals. In: Fink-Gremmels J, editor. *Animal Feed Contamination.* Cambridge, UK: Woodhead Publishing (2012). p.66-93.

Lunestad BT, Nesse L, Lassen J, Svihus B, Nesbakken T, Fossum K, et al. *Salmonella* in fish feed; occurrence and implications for fish and human health in Norway. *Aquaculture* (2007) 265:1-8. doi: 10.1016/j.aquaculture.2007.02.011.

Malorny B, Löfström C, Wagner M, Krämer N, Hoorfar J. Enumeration of *Salmonella* bacteria in food and feed samples by real-time PCR for quantitative microbial risk assessment. *Appl Environ Microbiol*. (2008) 74:1299-304. doi: 10.1128/AEM.02489-07.

Milanov DS, Prunić BZ, Velhner MJ, Pajić ML, Čabarkapa IS. RDAR morphotype: A resting stage of some *Enteroba cteriaceae*. *Food Feed Res.* (2015) 42:43-50. doi:10.5937/FFR1501043M.

Mooijman KA. The new ISO 6579-1: A real horizontal standard for detection of *Salmonella*, at last!. *Food Microbiol.* (2018) 71:2-7. doi: 10.1016/j.fm.2017.03.001.

Poggi-Varaldo HM. Agricultural wastes. *Water Environ Res.* (1999) 71:737-85. doi: 10.2175/106143099X133767.

Renggli F. Preventing an on-farm infection through proper feed treatment. *World Poultry Misset Salmonella Special* (1996) 12:34-5.

Richardson K, Weiss D. Control of *Salmonella* and other enteropathogens in animal feed. In *Proceedings of Forty-Ninth Western Poultry Disease Conference*. (2000). https://aaap.memberclicks.net/assets/WPDC/wpdcproceedings_2000.pdf [Accessed May 20, 2021].

The PEW Charitable Trusts. Food safety from farm to fork (2017). https://www.pewtrusts.org/-/media/assets/2017/07/food-safety-from-farm-to-fork-final.pdf [Accessed May 20, 2021].

Usman BA, Diarra SS. Prevalent diseases and mortality in egg type layers: An overview. *Int J Poult Sci*. (2008) *7*:304-10. doi: 10.3923/ijps.2008.304.310.

Guideline Documents:

Butcher GD, Miles RD. *Minimizing microbial contamination in feed mills producing poultry feed*. University of Florida Cooperative Extension Service, Institute of Food and Agriculture Sciences, EDIS (1995).

Domesle KJ, Young SR, Yang Q, Ge B. Loop-Mediated Isothermal Amplification for Screening *Salmonella* in Animal Food and Confirming Salmonella from Culture Isolation. *JoVE* (2020) 159:e61239. doi: 10.3791/61239.

Douglas J. *Production of salmonella-free turkey feed*. Kitchener, Canada: Hybrid Turkeys, A division of Nutreco Canada Inc. (2000).

European Food Safety Authority (EFSA). Data dictionaries/guidelines for reporting data on zoonoses, antimicrobial resistance and food-borne outbreaks using the EFSA Data models for the Data Collection Framework (DCF) in the Reporting Year 2012. *EFSA Supporting Publications* (2013) 10:409E. doi: 10.2903/sp.efsa.2013.EN-409.

European Food Safety Authority (EFSA). Technical specifications for the pilot on the collection of data on molecular testing of food-borne pathogens from food, feed and animal samples. *EFSA Supporting Publications* (2014) 11:712E. doi: 10.2903/sp.efsa.2014.EN-712.

European Food Safety Authority (EFSA), Delfino A, Pasinato L, Rizzi V, Stoicescu AV. Guidelines for reporting molecular typing data through EFSA's Data Collection Framework. *EFSA Supporting Publications* (2019) 16:1534E. doi: 10.2903/sp.efsa.2019.EN-1534.

European Food Safety Authority (EFSA), Jacobs W, Kuiling S, van der Zwaluw K. Molecular typing of Salmonella strains isolated from food, feed and animals: state of play and standard operating procedures for pulsed field gel electrophoresis (PFGE) and Multiple-Locus Variable number tandem repeat Analysis (MLVA) typing, profiles interpretation and curation. *EFSA Supporting Publications* (2014) 11:703E. doi: 10.2903/sp.efsa.2014.EN-703.

Risk Assessments:

European Food Safety Authority (EFSA) Panel on Biological Hazards (BIOHAZ). Scientific opinion on a quantitative microbiological risk assessment of *Salmonella* in slaughter and breeder pigs. *EFSA Journal* (2010) 8:1547. doi: 10.2903/j.efsa.2010.1547.

Hald T, Wingstrand A, Brøndsted T, Wong DMALF. Human health impact of *Salmonella* contamination in imported soybean products: a semiquantitative risk assessment. *Foodbourne Pathog Dis.* (2006) 3:422-31. doi: 10.1089/fpd.2006.3.422.

Rönnqvist M, Välttilä V, Ranta J, Tuominen P. *Salmonella* risk to consumers via pork is related to the Salmonella prevalence in pig feed. *Food Microbiol.* (2018) 71:93-7. doi: 10.1016/j.fm.2017.03.017.

Sauli I, Danuser J, Geeraerd AH, Van Impe JF, Rüfenacht J, Bissig-Choisat B, et al. Estimating the probability and level of contamination with *Salmonella* of feed for finishing pigs produced in Switzerland - the impact of the production pathway. *Int j Food Microbiol.* (2005) 100:289-310. doi: 10.1016/j.ijfoodmicro.2004.10.026.

Systematic Reviews and / or Meta-analyses:

Hald T, Wingstrand A, Pires SM, Vieira A, Domingues AR, Lundsby K, et al. Assessment of the human-health impact of *Salmonella* in animal feed. Soborg: DTU Food (2012). 76p.

Wilhelm B, Rajić A, Parker S, Waddell L, Sanchez J, Fazi, A, et al. Assessment of the efficacy and quality of evidence for five on-farm interventions for *Salmonella* reduction in grow-finish swine: a systematic review and meta-analysis. *Prev Vet Med.* (2012) 107:1-20. doi: 10.1016/j.prevetmed.2012.07.011.

***In silico* Models:**

Crabb HK, Allen JL, Devlin JM, Firestone SM, Stevenson MA, Gilkerson JR. The use of social network analysis to examine the transmission of *Salmonella spp.* within a vertically integrated broiler enterprise. *Food Microbiol.* (2018) 71:73-81. doi: 10.1016/j.fm.2017.03.008.

Daniels MJ, Hutchings MR, Greig A. The risk of disease transmission to livestock posed by contamination of farm stored feed by wildlife excreta. *Epidemiol Infect.* (2003) 130:561-68. doi: 10.1017/S0950268803008483.

European Food Safety Authority (EFSA). Development of web monitoring systems for the detection of emerging risks. *EFSA Journal* (2009) *7*:1355. doi: 10.2903/j.efsa.2009.1355.

Hill AA, Simons RR, Kelly L, Snary EL. A farm transmission model for *Salmonella* in pigs, applicable to EU Member States. *Risk Anal.* (2016) 36:461-81. doi: 10.1111/risa.12356.

Krieter J. Evaluation of *Salmonella* surveillance in pigs using a stochastic simulation model. *Arch Anim Breed.* (2004) 47:337-49. doi:10.5194/aab-47-337-2004.

Niemi JK, Heinola K, Simola M, Tuominen P. *Salmonella* control programme of pig feeds is financially beneficial in Finland. *Front Vet Sci.* (2019) 6:200. doi: 10.3389/fvets.2019.00200.

Okelo PO, Wagner DD, Carr LE, Wheaton FW, Douglass LW, Joseph SW. Optimization of extrusion conditions for elimination of mesophilic bacteria during thermal processing of animal feed mash. *Anim Feed Sci Technol.* (2006) 129:116-37. doi: 10.1016/j.anifeedsci.2005.12.011.

Trinetta V, Magossi G, Allard MW, Tallent SM, Brown EW, Lomonaco S. Characterization of *Salmonella enterica* isolates from selected US swine feed mills by whole-genome sequencing. *Foodborne Pathog Dis.* (2020) 17:126-36. doi: 10.1089/fpd.2019.2701.

Välttilä V, Ranta J, Rönnqvist M, Tuominen P. Bayesian model for tracing *Salmonella* contamination in the pig feed chain. *Food Microbiol.* (2018) 71:82-92. doi: 10.1016/j.fm.2017.04.017.

Weigel RM, Nucera D, Qiao B, Teferedegne B, Suh D., Barber DA, et al. Testing an ecological model for transmission of *Salmonella enterica* in swine production ecosystems using genotyping data. *Prev Vet Med.* (2007) 81:274-89. doi: 10.1016/j.prevetmed.2007.04.020.

Appendix 3: **Details of feedstuffs included in the single-ingredient category, as defined by authors, in the scoping review on *Salmonella* in animal feeds.**

Manufacturing plant sector

Grains and oilseeds	Grain by-products	Plant protein products	Animal by-products	Recycled food products	Other
barley	brewers	alfalfa meal	animal by-products	milk products	algae waste
broll		canola meal	blood meal	recycled food products	biscuit flour
corn products		corn gluten	bone meal	vegetable feeds	citrus pulp
dry grain		cottonseed meal	chicken meal	whey	cocoa bean shell
hominy		grits	eggshell flour		coconut
maize		linseed meal	feather meal		egg powder
mash		meal	meat and bone meal		flour
oilseed byproducts		oil meal	meat meal		insects
rapeseed		palm kernel meal	slaughterhouse sludge		maize flour
rice bran		rapeseed meal			poultry litter
sorghum		soybean meal			poultry waste
soy		sunflower meal			sugarbeet pulp
soya					tapioca
soya oil					
wet grain					
wheat bran					
wheat grain					

Transportation sector

Grains and oilseeds	Grain by-products	Plant protein products	Animal by-products	Recycled food products	Other
corn		soybean meal	animal by-products		milk powder
			bonemeal		
			feather meal		
			fish meal		
			meat and bone meal		

Retail sector

Grains and oilseeds	Grain by-products	Plant protein products	Animal by-products	Recycled food products	Other
cottonseed		soybean meal	animal by-products		milk replacer
maize			fish meal		
mash					
peanut					
rapeseed					
safflower					
sunflower					

On-farm sector

Grains and oilseeds	Grain by-products	Plant protein products	Animal by-products	Recycled food products	Other
canola	brewers	cassava meal	animal byproducts	bakery meal	banana
cassava	corn distillers solubles	corn gluten	clam meat	buttermilk	beet pulp
corn	dry byproduct	corn meal	colostrum	recycled food products	duck weed
corn / barley mix	wet byproduct	soybean meal	egg shells	vegetable oil	extruded swine feed
corn bran			fish meal	whey	grass seed
cottonseed			hatchery waste eggs	yoghurt	liquid swine feed

dry grain			poultry meal		milk replacer
grain			poultry waste		molasses
maize			waste milk		organic mixed ration
mash					pea flour
mixed grain					sugar syrup
oil seeds					taro
organic grains					
rapeseed					
rice					
wet grain					
wheat					

Sector not specified

**Grains and oilseeds**	**Grain by-products**	**Plant protein products**	**Animal by-products**	**Recycled food products**	**Other**
barley	brewers	canola meal	animal by-products	bread	citrus pulp
cereal		corn gluten	animal fat	recycled food products	clamshell
corn		corn grits	blood meal	vegetable material	cocoa
corn bran		corn meal	blood meal and feathers		dry coffee pulp
cotton		cottonseed meal	bone meal		game bird feed
cottonseed		oilseed meal	colostrum		insects
dry grain		palm kernel meal	dairy products		maize flour
ground milk thistle seeds		potato protein	egg		milk replacer
linseed		rapemeal	fish oil		molasses
maise / maize		rapeseed meal	fish powder		papaya latex

mash	soybean meal	fish products		ratite (emu) starter-crumble
millet	sunflower meal	frog waste meal		sewage as feed
oats	wheat meal	greaves and poultry offal meal		sugar cane molasses
oilseeds		meat and bone meal		sugarbeet pulp
olive cake		meat meal		wheat flour
palm kernel		meat products		
peanut		milk		
peas		rumen contents		
pulses		spent hen meal		
rapeseed		viscera meal		
rice				
rice bran				
rye				
screenings				
sorghum				
soya				
soya expeller				
sugar cane mash				
sunflower				
triticale				
wet grain				
wheat				
wheat bran				

Appendix 4: **Serovars identified in the scoping review of *Salmonella* in animal feeds (number of studies reporting each serovar), by sector of animal feed production.**

Field

Typhimurium	2
Typhimurium DT140	1
Anatum	1
Derby	1
Durham	1
Kedougou	1
Mbandaka	1
Montevideo	1
Newport	1
Rissen	1
Senftenberg	1
Stanley	1

Plant

Senftenberg	37
Typhimurium	37
Agona	30
Mbandaka	30
Montevideo	26
Infantis	25
Enteritidis	23
Anatum	20
Schwarzengrund	18
Livingstone	16
Tennessee	16
Havana	15
Cubana	13
Ohio	13
Rissen	13
Derby	12
Newport	12
Kentucky	10
Lexington	10
Cerro	9
Give	9
Muenster	9
Orion	9
Worthington	9

Oranienburg	9
Braenderup	8
Bareilly	7
Bredeney	7
Kendougou	7
Agama	6
Amsterdam	6
Hadar	6
Heidelberg	6
Meleagridis	6
Saintpaul	6
4,12:d:-	5
I 4,[5],12:I:-	5
Aberdeen	5
Alachua	5
Brandenburg	5
Idikan	5
Johannesburg	5
Lille	5
Llandoff	5
London	5
Yoruba	5
Adelaide	4
Bere	4
California	4
Indiana	4
Kottbus	4
Muenchen	4
Muenchen	4
Ruiru	4
Virchow	4
Ajiobo	3
Albany	3
Bergen	3
Berta	3
Binza	3
Bovismorbificans	3
Dusseldorf =2	3
Goldcoast	3
Ibadan	3
Isangi	3

Java	3
Jerusalem	3
Kiambu	3
Kingston	3
Liverpool	3
Molade	3
Morehead	3
Ouakam	3
Poona	3
Reading	3
Soerenga	3
Thompson	3
Weltevreden	3
I 6,7	2
Abony	2
Altona	2
Amager	2
Banana	2
Blockley	2
Chester	2
Corvallis	2
Drypool	2
Dublin	2
Durham	2
Ealing	2
Fremantle	2
Gaminare	2
Gloucester	2
Hartford	2
Javiana	2
Kapemba	2
Kibi	2
Lansing	2
Madelia	2
Manhattan	2
Minnesota	2
Oslo	2
Panama	2
Paratyphi B	2
Putten	2
Sandiego	2

Schleissheim	2
Singapore	2
Stourbridge	2
Taksony	2
Tees	2
Tilburg	2
Vejle	2
Westhampton	2
1 G.O.:7	1
1,3,19:i:-	1
4:d:-	1
6,7:z:1,5	1
8,2O:i:-	1
13,19: non-motile	1
13,23:z37:-	1
16:-:-	1
16,7;1.5;2 efnat	1
I 3,10:--:1,6	1
I 4,12:--:--	1
I 6,7;d;2 efnat	1
I (6) 8;210;2 efnat	1
I (6) 14;a;2 efnat	1
IIIa. 42:z4,z23	1
O:3,10	1
O:3,10:eh:-	1
O:6,7	1
O:6,8	1
O:13,23	1
O:16,:c:-	1
S6,7.-.-	1
S6,7.k.-	1
Aarhus	1
Aba	1
Abaetetuba	1
Abortus Bovis	1
Adabraka	1
Aflao	1
Alabama	1
Alamo	1
Albanyl	1
Alsterdorf	1
Arechavaleta	1
Arkansas	1
Azteka	1
Babelsberg	1
Bahrenfeld	1
Ball	1
Bardo	1
Barranquilla	1
Be	1
Beaudesert	1
Beitri	1
Benefica	1
Bochum	1
Bonariensis	1
Brancaster	1
Brazzavile	1
Breda	1
Brezany	1
Broughton	1
Caminara	1
Cannobio	1
Carrau	1
Chailey	1
Champaign	1
Chandans	1
Chao	1
Charity	1
Chartres	1
Chincol	1
Clackamas	1
Cocvafvils	1
Coeln	1
Colorado	1
Dahra	1
Dallgow	1
Demerara	1
Diarizonae	1
Djermaia	1
Djugu	1
Donna	1
Emek	1
Eppendorf	1
Eschweiler	1
Etterbeek	1
Fischerkietz	1
Florida	1
Freetown	1
Friednau	1
Gabon	1
Gallinarum	1
Gatow	1
Gatuni	1
Georgia	1
Glostrup	1
Glowcester	1
Hillington	1
Hissar	1
Hithergreen	1
Houtenae 51:a	1
Hvittingfoss	1
Irachau	1
Kaapstad	1
Kambok	1
Kinondoni	1
Kortrijk	1
Krefeld	1
Kunduchi	1
Lagos	1
Lamberhurs	1
Langeveld	1
Leno	1
Leopoldville	1
Limete	1
Manchester	1
Mandoff	1
Mango	1
Maricopa	1
Menston	1
Miami	1
Mons	1
Morillons	1
Newbrunswick	1
Newlands	1
Nitra	1
Norwich	1
Obogu	1
Ohlstedt	1
Okatie	1
Onderstepoort	1
Orientalis	1
Othamarschen	1
Ouakum	1
Pakistan	1
Paratyphi A	1

Paratyphi B Fagt. Dundee	1
Paratyphi BV Java	1
Paratyphi C	1
Parkroyal	1
Petahtikve	1
Ploufragan	1
Plymouth	1
Pomona	1
Potsdam	1
Regent	1
Rough	1
Ruiri	1
Salamae	1
Salford	1
Saloniki	1
Sambre	1
Sangera	1
Saphra	1
Sarajane	1
Schoeneberg	1
Seegefeld	1
Sinstorf	1
Slade	1
Somone	1
Southbank	1
Stanley	1
Stockholm	1
Sundsvall	1
Svedvi	1
Tabligo	1
Tallahasse	1
Thomasville	1
Tournai	1
Uganda	1
Urbana	1
Vaertan	1
Wangata	1
Wagenia	1
Wandsbek	1
Warragul	1
Waycross	1
Westphalia	1
Wien	1
Wil	1

Winston	1
Zanzibar	1
BO-Group (as described by authors)	1
CO-group (as described by authors)	1
EO-Group (as described by authors)	1
Group C1 (as described by authors)	1

Transportation

Infantis	4
Livingstone	4
Mbandaka	4
Oranienburg	4
Senftenberg	4
Montevideo	3
Ohio	3
Orion	3
Rissen	3
Schwarzengrund	3
Tennessee	3
Amager	2
Anatum	2
Bareilly	2
Brandenburg	2
Derby	2
Enteritidis	2
Havana	2
Heidelberg	2
London	2
Muenster	2
Tilburg =1	2
Westhampton	2
II (as described by authors)	2
untypable 6,7	1
Aberdeen	1
Adelaide	1
Agona	1
Arkansas	1
Babelsberg	1
Binza	1
Beitri	1
Blockley	1
Braenderup	1
Brazzaville	1
Bredeney	1
Chester	1
Clackamas	1
Corvallis	1
Cubana	1
Donna	1
Dublin	1
Edinburgh	1

Emek	1
Fresno	1
Give	1
Goldcoast	1
Grampian	1
Grostrup	1
Hadar	1
Hato	1
Hillington	1
Hindmarsh	1
Indiana	1
Irion	1
Isangi	1
Johannesburg	1
Kambole	1
Kambok	1
Kapemba	1
Kentucky	1
Kisii	1
Krefeld	1
Leopoldville	1
Lexington	1
Liverpool	1
Mons	1
Natal	1
Ouakam	1
Othamarschen	1
Regent	1
Roterberg	1
Rough	1
Saintpaul	1
Schleissheim	1
Siegburg	1
Sinstorf	1
Suberu	1
Thomasville	1
Thompson	1
Typhimurium	1
Urbana	1
UT (as described by authors)	1
Worthington	1

BO-group (as described by authors)	1
CO-group (as described by authors)	1
EO-group (as described by authors)	1

Retail

Enteritidis	3
Newport	3
Cubana	2
Typhimurium	2
Bareilley	1
Choleraesuis	1
Heidelberg	1
Infantis	1
Javiana	1
Kentucky	1
Mbandaka	1
Montevideo	1
Senftenberg	1

Farm

Typhimurium	32
Enteritidis	26
Senftenberg	18
Mbandaka	17
Infantis	14
Derby	11
Worthington	11
Anatum	10
Kentucky	10
Montevideo	8
Agona	7
Newport	7
Tennessee	7
Heidelberg	6
Braenderup	5
Meleagridis	5
Orion	5
Cerro	4
Havana	4
Livingstone	4
Muenster	4
Oranienburg	4
Schwarzengrund	4
Stanley	4
Thompson	4
Albany	3
Bredeney	3
Cubana	3
Java	3
Lexington	3
Muenchen	3
Ohio	3
Rissen	3
Rough	3
Virchow	3
Weltevreden	3
1,4,[5],12:i:-	2
Brandenburg	2
Copenhagen	2
Corvallis	2
Gallinarum	2
Give	2
Hadar	2
Javiana	2
Johannesburg	2
Kottbus	2
Newington	2
Panama	2
Saintpaul	2
Thomasville	2
Urbana	2
4, 12: i:-	1
13,19: non-motile	1
O:4,5:-:1,2	1
OUT:d:1,7	1
OUT:r:1,5	1
II (as described by authors)	1
Adabraka	1
Altona	1
Amersfoort	1
Amsterdam	1
Arkansas	1
Bareilly	1
Berta	1
Binza	1
Blockley	1
Bovismorbificans	1
Broughton	1
Concord	1
Dublin	1
Durham	1
Ealing	1
Eastbourne	1
Emek	1
Fresno	1
Georgia	1
Hvittingfoss	1
Inverness	1
Isangi	1
Jerusalem	1
Krefeld	1
Lille	1
London	1
Manhatten	1
Paratyphi B biovar Java	1
Poona	1
Pullorum	1
Reading	1
Rough:gms:-	1
Rough:z:r6	1
Rubislaw	1
Sandiego	1
Simsbury	1
Slade	1
Suberu	1
Takoradi	1
Taksony	1
Typhi	1
Uganda	1
Yovokome	1

Sector not specified

Typhimurium	75
Enteritidis	47
Senftenberg	25
Agona	23
Infantis	21
Mbandaka	15
Montevideo	15
Tennessee	15
Livingstone	14
Anatum	12
Heidelberg	9
Cerro	8
Schwarzengrund	8
Kentucky	7
Rissen	7
Cubana	6
Derby	6
Gallinarum	5
Hadar	5
Havana	5
Newport	5
Ohio	5
Oranienburg	5
Virchow	5
Alachua	4
Braenderup	4
Meleagridis	4
Muenster	4
Orion	4
Pullorum	4

Binza	3
Choleraesuis	3
Dublin	3
Gaminara	3
Give	3
Johannesburg	3
Lexington	3
Lille	3
Reading	3
Worthington	3
I 4,[5],12-i-	2
Berta	2
Bredeney	2
California	2
Ealing	2
Jerusalem	2
Kedougou	2
Litchfield	2
Liverpool	2
Newington	2
Newlands	2
Pomona	2
Putten	2
Rough	2
Ruiru	2
Soerenga	2
Stockholm	2
Thompson	2
Typhi	2
Uganda	2
Yoruba	2
I 4,[5],12:i:-	1
I 6,7::1,5	1
I 6,7:k:	1
II (as described by authors)	1
3,19::z27	1
4:b:-	1
4:12:d:	1
4, 12:d:-	1
6,7::1,5	1
6,7:b:-	1
6,7:d	1
8,20:poorly motile	1
21::e,n,x	1

42:z4,z23	1
50.D-Z8	1
212:nonmotile	1
B gr. (04) (as described by authors)	1
C gr. (07) (as described by authors)	1
R form (as described by authors)	1
Adelaide	1
Agama	1
Amager	1
Amsterdam	1
Arizona	1
Arkansas	1
Bareilly	1
Barranquilla	1
Bere	1
Bergen	1
Boecker	1
Brandenburg	1
Coeln	1
Djugu	1
Driffield	1
Drypool	1
Eimsbuettel	1
Emek	1
Eschweiler	1
Gafsa	1
Gera	1
Grampian	1
Haardt	1
Hvittingfoss	1
Ibadan	1
Idikan	1
Indiana	1
Irumu	1
Isangi	1
Java	1
Jodhpur	1
Kiambu	1
Kingston	1

Kivu	1
Lamphun	1
Malstatt	1
Michigan	1
Minneapolis	1
Mokola	1
Molade	1
Morningside	1
Muenchen	1
Nakuru	1
Ohlstedt	1
Othmarschen	1
Ouakam	1
Panama	1
Paratyphoids (as described by authors)	1
Reubeuss	1
Roodepoort	1
Rubislaw	1
Saintpaul	1
Stanley	1
Stanleyville	1
Taksony	1
Thomasville	1
Urbana	1
Weltevreden	1

Appendix 5: **List of the 20 most common *Salmonella* serovars associated with human illness in the US and EU, the five most commonly associated with illness in each animal species in the US, and the five most commonly found in food from animals intended for human consumption in each animal species in the US.**

**Illness in Humans, EU[1]**	**Illness in Humans, US[2]**	**Illness in Animals, US[3]**	**Food Intended for Human Consumption, US[4]**
Enteritidis *	Enteritidis *[4]	Enteritidis (chicken) *	Kentucky (chicken) *
Typhimurium *	Newport *	Typhimurium (chicken) *	Infantis (chicken) *
I 4,[5],12:i:-	Typhimurium *	Kentucky (chicken) *	Enteritidis(chicken) *
Infantis *	Javiana *	Heidelberg (chicken) *	Schwarzengrund (chicken) *
Newport *	I 4,[5],12:i:-	III 13,23:g,z51:- (chicken)	Typhimurium (chicken) *
Derby *	Infantis *	Senftenberg (turkey) *	Reading (turkey)
Stanley *	Saintpaul	Ouakam(turkey)	Schwarzengrund (turkey) *
Kentucky *	Braedenrup	Bredeney (turkey)	Uganda (turkey)
Napoli	Muenchen	Albany (turkey)	Infantis (turkey) *
Agona *	Montevideo *	Typhimurium (turkey) *	Agona (turkey) *
Virchow	Thompson	4,[5],12:i- (pig)	Anatum (pork) *
Coeln	I 13,23:b:-	Typhimurium (pig) *	4,[5],12:i:- (pork)
Bovismorbificans	Mississippi	Derby (pig) *	Infantis (pork) *
Java	Oranienburg *	Infantis (pig) *	Derby (pork) *
Mikawasima	Typhi	Agona (pig) *	Adelaide (pork)
Chester	Rough/Nm *	Dublin (cattle)	Montevideo (beef) *
Bareilly *	Agona *	Cerro (cattle)	Muenchen (beef)
Saintpaul	Paratyphi B var. L(+) tartrate+ (Java)*	Typhimurium (cattle) *	Anatum (beef) *
Branderup	Anatum *	Montevideo (cattle) *	Newport (beef) *
Hadar	Reading	Heidelberg (cattle) *	Dublin (beef)

[1] The European Union One Health 2019 Zoonoses Report, Table 15: Top 20 Distribution of reported confirmed cases of human salmonellosis in the EU/EEA, 2017-2019, by the 20 most frequent serovars in 2019 (EFSA and ECDPC, 2021)

[2] Center for Disease Control, FoodNet Fast, Pathogen Surveillance – *Salmonella*; Distribution of Salmonella infections in 2019, Percentage of infections by serotype – FoodNet sites (available at https://www.cdc.gov)
[3] Center for Disease Control. *Salmonella* Serotypes Isolated from Animals and Related Sources, January 1-December 31, 2016, (2016b) Available at https://www.cdc.gov/nationalsurveillance/pdfs/salmonella-serotypes-isolated-animals-and-related-sources-508.pdf
[4] USDA, Food Safety and Inspection Service: Quarterly Sampling Reports on Salmonella, Fiscal Year 2019. (available at https://www.fsis.usda.gov/science-data/data-sets-visualizations/microbiology/microbiological-testing-program-rte-meat-and-7)
* *Salmonella* serovars with * are amongst the 10 most frequently investigated in a sector of feed production animal feeds, feed equipment, or feed environment in the scoping review of *Salmonella* in animal feeds.
